# Prospective Study on Retinal Nerve Fibre Layer Thickness Changes in Isolated Unilateral Retrobulbar Optic Neuritis

**DOI:** 10.1155/2013/694613

**Published:** 2013-12-25

**Authors:** Gordon S. K. Yau, Jacky W. Y. Lee, Patrick P. K. Lau, Victor T. Y. Tam, Winnie W. Y. Wong, Can Y. F. Yuen

**Affiliations:** ^1^Department of Ophthalmology, Caritas Medical Centre, 111 Wing Hong Street, Kowloon, Hong Kong; ^2^Department of Medicine and Geriatric, Caritas Medical Centre, 111 Wing Hong Street, Kowloon, Hong Kong

## Abstract

*Purpose.* To investigate the retinal nerve fibre layer (RNFL) thickness after unilateral acute optic neuritis using optical coherence tomography (OCT). *Patients and Methods*. This prospective cohort study recruited consecutive patients with a first episode of isolated, unilateral acute optic neuritis. RNFL thickness and visual acuity (VA) of the attack and normal fellow eye were measured at presentation and 3 months in both the treatment and nontreatment groups. *Results.* 11 subjects received systemic steroids and 9 were treated conservatively. The baseline RNFL thickness was similar in the attack and fellow eye (*P* ≥ 0.4). At 3 months, the attack eye had a thinner temporal (*P* = 0.02) and average (*P* = 0.05) RNFL compared to the fellow eye. At 3 months, the attack eye had significant RNFL thinning in the 4 quadrants and average thickness (*P* ≤ 0.0002) compared to baseline. The RNFL thickness between the treatment and nontreatment groups was similar at baseline and 3 months (*P* ≥ 0.1). Treatment offered better VA at 3 months (0.1 ± 0.2 versus 0.3 ± 0.2 LogMAR, *P* = 0.04). *Conclusion*. Generalized RNFL thinning occurred at 3 months after a first episode of acute optic neuritis most significantly in the temporal quadrant and average thickness. Visual improvement with treatment was independent of RNFL thickness.

## 1. Introduction

Optic neuritis is a demyelinating inflammation of the optic nerve characterised by a sudden onset of vision loss and dyschromatopsia associated with ocular pain [1]. Postinflammatory changes include axonal loss and optic nerve atrophy [2].

Optical coherence tomography (OCT) is a noncontact imaging device used in ophthalmology to provide high resolution cross-sectional images and quantification of the ocular structures, namely, the macula, optic nerve head, and retinal nerve fibre layer (RNFL).

In recent years, neuroophthalmologists recommended use of OCT for objective morphological assessment and quantification of optic nerve head and peripapillary RNFL thickness [3]. Thus, structural axonal loss in the optic nerve can be quantified by measuring the peripapillary RNFL thickness using OCT [4]. Measuring the RNFL thickness after an acute episode of optic neuritis provides a structural assessment of the optic nerve, enables prognostic evaluations, and can also help to differentiate between neuromyelitis optica (NMO) and multiple sclerosis (MS) [5, 6].

The aim of this study was to assess the changes in RNFL after a first attack of optic neuritis and to investigate the impact of treatment on axonal loss.

## 2. Patients and Methods

The study was approved by the Institutional Review Board of the Hospital Authority of Hong Kong and informed consent was obtained prior to commencement of the study. The study was conducted in accordance with the Declaration of Helsinki.

This is a prospective cohort study conducted by Caritas Medical Centre, Hong Kong between October 2010 and December 2012. Consecutive, consenting adults, 18–80 years of age, diagnosed with a first episode of isolated unilateral acute (<8 days of symptoms) optic neuritis were recruited and followed up for at least 3 months. The diagnosis of optic neuritis was made based on a compatible history involving ocular pain; clinical examination demonstrating impairment of optic nerve function including dyschromatopsia, visual acuity (VA) impairment, optic disc swelling, or relative afferent papillary defect; andthe absence of any space occupying lesion on a computed tomography scan of the brain and orbit.

All subjects underwent neuroophthalmic examination by an ophthalmologist (SKY) and a neurologist (PKL). All subjects underwent imaging for RNFL thickness via a spectral-domain OCT (Heidelberg Engineering, CA, USA).

Contrast magnetic resonance image (MRI) scan of the brain and orbit and blood taking for autoimmune markers and aquaporin-4 antibody were subsequently performed to rule out other causes of optic neuritis. Subjects with known or confirmed diagnosis of MS, NMO, autoimmune disease, other optic neuropathies, and glaucoma were excluded.

Subjects with a high visual demand requiring a hastened visual recovery, were administered a systemic steroid treatment (intravenous methyl-prednisolone 250 mg every 6 hours for 3 days, followed by oral prednisone 1 mg/kg/day for 11 days) as per the Optic Neuritis Treatment Trial (ONTT) protocol [7] after explanation of the risks and benefits of treatment (treatment group). The nontreatment group did not receive any active medical treatment.

Best corrected Snellen VA was measured at baseline and 3 months and converted to LogMAR for statistical analysis.

### 2.1. Measurement of the Peripapillary RNFL Thickness with OCT

The Spectralis OCT (Heidelberg Engineering, CA, USA) for RNFL was operated by a trained imaging technician who was masked to subjects' diagnosis. RNFL thickness was measured around the optic nerve head obtained using the fast RNFL thickness protocol. Scans were repeated 3 times and assessed for signal strength and centration. Scans with signal strength quality *Q* ≤ 16 [HS] or poor centration were excluded. RNFL thickness was analyzed with the RNFL Single Exam Report OU with fovea-to-disc technology. The RNFL thickness of each of the 4 quadrants and the average RNFL were recorded for the attacked and nonattack fellow eye at baseline (on presentation) and at 3 months after the attack in all subjects (including both the treatment and nontreatment groups).

### 2.2. Statistics

The Mann-Whitney *U* test was used to assess differences in the mean RNFL for the following:

- treatment versus non treatment eye at baseline,
- attack versus fellow eye at baseline,
- attack versus fellow eye at 3 months after attack,
- treatment versus non treatment eye at 3 months after attack,
- VA in treatment group and nontreatment group at baseline,
- VA in treatment group and nontreatment group at 3 months.

The Wilcoxon signed rank test was used to assess differences in the mean RNFL for the following:

- attack eye at baseline versus 3 months,
- fellow eye at baseline versus 3 months.

The correlation between VA and average RNFL thickness at 3 months was determined by Spearman's rank correlation coefficient.

Means were expressed as mean ± standard deviation and statistical significance was defined as *P* < 0.05.

## 3. Results

Twenty patients were included in the study. All subjects had a first episode of an isolated, unilateral optic neuritis. Female subjects comprised of 75% of the study population (male : female ratio was 5 : 15). There were 11 right eyes and 9 left eyes. The mean age was 34.8 ± 12.1 years. All subjects were ethnic Chinese. Eleven patients were in the treatment group and 9 patients did not receive any treatment. There were no complications from the systemic steroid treatment.

In all 20 subjects, the baseline quadrant and average RNFL thickness were statistically similar at baseline in the attack and nonattack fellow eye (*P* ≥ 0.4). Likewise, the baseline quadrant and average RNFL thickness were similar in both the treatment and nontreatment groups (*P* ≥ 0.1) (Table 1).

At 3 months after attack, there was a thinner temporal (*P* = 0.02) and average (*P* = 0.05) RNFL thickness compared to the nonattack fellow eye (Table 2). On comparison of the RNFL thickness between the treatment and nontreatment group at 3 months, there was no significant difference in the quadrant or average thickness (*P* ≥ 0.3).

At 3 months after attack, there was a significant RNFL thinning in the superior (*P* = 0.0002), nasal (*P* = 0.0001), inferior (*P* = 0.0001), temporal (*P* = 0.0001), and average thickness (*P* = 0.0001) in the attack eye when compared to its baseline values (Table 3). There was no change in RNFL in the nonattack fellow eye at baseline and 3 months.

The treatment (0.6 ± 0.4 LogMAR) and nontreatment (0.7 ± 0.3 LogMAR) groups had comparable baseline VA (*P* = 0.6). The time to achieve a 3-line improvement of LogMAR VA from baseline was 13 days and 18 days in the treatment and nontreatment group, respectively. At 3 months after attack, the treatment group had a better VA (0.1 ± 0.2 LogMAR) than the nontreatment group (0.3 ± 0.2 LogMAR) (*P* = 0.04). There was no correlation between the VA and average RNFL thickness at 3 months (*P* = 0.2).

## 4. Discussion

Axonal degeneration and optic nerve atrophy after an episode of optic nerve inflammation have been described in idiopathic optic neuritis, MS, and NMO. This is one of the few studies in the literature that objectively quantify the RNFL thickness after an episode of optic neuritis in the Chinese population.

Whilst OCT is a powerful tool for the assessment of the RNFL, different OCT machines employ different acquisition technologies and data analysis software; thus it has been shown that significant variability in RNFL thickness can exist among different OCT machines [8]. To alleviate this problem, all patients in our series were examined with a single machine with the same software version by a single masked operator.

Previous literatures demonstrated that there was temporal quadrant thinning of the RNFL after an episode of optic neuritis [9, 10] and that the RNFL thickness progressively decreased despite improvement of visual function [11]. In our study, we demonstrated a progressive RNFL thinning in all 4 quadrants as well as the average RNFL thickness (all *P* < 0.0002) in the attack eye at 3 months after a first attack of optic neuritis. Our findings were consistent with that of Pro et al. that reported temporal RNFL thinning at 3 months after optic neuritis [9]. In our series, we found that the temporal (*P* = 0.02) and average RNFL (*P* = 0.05) were most significantly thinned in the attack eye at 3 months after the attack when compared to the normal fellow eye. This suggests that the temporal quadrant may be the first to be affected by optic neuritis. The RNFL in the other quadrants was also thinner in the attack eye at 3 months compared to the fellow eye but was just short of reaching statistical significance.

The ONTT study demonstrated that the treatment with intravenous followed by oral steroids speeded up the recovery of visual function in the short term with no significant difference in the final visual outcome [7, 12]. Our findings were consistent with the ONTT by demonstrating that the treatment group took less time (13 days) to achieve a 3-line improvement of LogMAR VA compared to the nontreatment group (18 days).

Gerling and Kommerell demonstrated that the high dose intravenous steroid treatment had beneficial effects up to 6 months [13]; similarly, we demonstrated that the treatment group still had a better VA compared to the nontreatment group at 3 months after the attack (*P* = 0.04). High dose steroid treatment could limit the extent of acute inflammation and the demyelination process. The remyelination process was shown to be related to the initial extent of demyelination [14]. Our study did not show that treatment improved the preservation of axonal cells or promoted early remyelination as evident by a statically similar RNFL at 3 months in the treatment and nontreatment group despite the improvement in VA. There was also no correlation between the VA and average RNFL at 3 months (*P* = 0.2). Thus, it seems that VA is independent of axonal thickness in the early phase of optic neuritis. Sühs et al. showed that both steroid and interferon-beta treatments had no effect on the preservation of RNFL thickness in isolated optic neuritis [15]. With the advancement in RNFL imaging technology, we are able to better understand the dynamic pathophysiology of optic neuritis. Measurements of axonal loss are currently one of the main outcome parameters for neuroprotection treatment trials for optic neuritis [16, 17].

This study had its limitations. Firstly, the assignment of treatment and nontreatment groups was not randomized, but the authors felt that it would be unethical to withhold treatment in those with a high visual demand when systemic steroids can hasten the visual recovery process. Secondly, a longer duration of followup would give us a better understanding of the long-term axonal status of optic neuritis in addition to the early results reported in this study. Thirdly, a study involving a large sample size would enable us to further stratify the different etiologies of optic neuritis including those associated with MS or NMO although such subclasses analyses were beyond the scope of this study.

In conclusion, RNFL thinning on OCT was observed in all 4 quadrants and in the average thickness at 3 months after a first attack of acute optic neuritis. The temporal quadrant and the average thickness were thinned most significantly compared to the normal fellow eye. Treatment with systemic steroids resulted in a better 3-month VA without any influence on the RNFL thickness.

## Figures and Tables

**Table 1:** Comparison of mean RNFL thickness.

	Superior RNFL	Nasal RNFL	Inferior RNFL	Temporal RNFL	Average RNFL
Baseline RNFL in the treatment versus nontreatment eye (*P* value)	1.0	0.5	0.8	0.1	0.6

Baseline RNFL in the attack versus fellow eye (*P* value)	0.8	0.4	0.9	0.5	0.8

3-month RNFL in the attack versus fellow eye (*P* value)	0.2	0.3	0.1	0.02*	0.05*

3-month RNFL in the treatment versus nontreatment eye (*P* value)	0.8	0.7	0.9	0.3	1.0

Attack eye RNFL at baseline versus 3 months (*P* value)	0.0002*	0.0001*	0.0001*	0.0001*	0.0001*

Fellow eye RNFL at baseline versus 3 months (*P* value)	0.1	0.5	0.1	0.4	0.3

*Statistical significance.

**Table 2:** RNFL thickness in the attack eye and nonattack fellow eye at 3 months.

	Superior RNFL	Nasal RNFL	Inferior RNFL	Temporal RNFL	Average RNFL
3-month RNFL thickness in the attack eye (*μ*m)	135.0 ± 8.6	82.7 ± 6.2	130.6 ± 8.2	67.15 ± 7.7	103.9 ± 6.0
3-month RNFL thickness in the fellow eye (*μ*m)	138.4 ± 7.0	84.7 ± 5.9	134.2 ± 6.6	73.8 ± 8.1	107.8 ± 5.7
*P* value	0.2	0.3	0.1	0.02*	0.05*

*Statistical significance.

**Table 3:** RNFL thickness in the attack eye at baseline and 3 months.

	Superior RNFL	Nasal RNFL	Inferior RNFL	Temporal RNFL	Average RNFL
Baseline RNFL thickness (*μ*m)	139.7 ± 8.2	87.3 ± 6.7	135.1 ± 8.0	71.6 ± 8.1	108.4 ± 5.9
3-month RNFL thickness (*μ*m)	135 ± 8.6	82.7 ± 6.2	130.6 ± 8.2	67.2 ± 7.7	103.9 ± 6.0
*P* value	0.0002*	0.0001*	0.0001*	0.0001*	0.0001*

*Statistical significance.
